# Identification and Functional Characterization of a Novel Monotreme- Specific Antibacterial Protein Expressed during Lactation

**DOI:** 10.1371/journal.pone.0053686

**Published:** 2013-01-09

**Authors:** Swathi Bisana, Satish Kumar, Peggy Rismiller, Stewart C. Nicol, Christophe Lefèvre, Kevin R. Nicholas, Julie A. Sharp

**Affiliations:** 1 Centre for Chemistry and Biotechnology, Deakin University, Geelong, Victoria, Australia; 2 Centre for Cellular and Molecular Biology, Council of Scientific and Industrial Research (CSIR), Hyderabad, Andhra Pradesh, India; 3 Anatomical Sciences, University of Adelaide, Adelaide, South Australia; 4 Pelican Lagoon Research and Wildlife Centre, Penneshaw, South Australia, Australia; 5 School of Zoology, University of Tasmania, Hobart, Tasmania, Australia; BiK-F Biodiversity and Climate Research Center, Germany

## Abstract

Monotremes are the only oviparous mammals and exhibit a fascinating combination of reptilian and mammalian characters. They represent a component of synapsidal reproduction by laying shelled eggs which are incubated outside the mother’s body. This is accompanied by a prototherian lactation process, marking them as representatives of early mammals. The only extant monotremes are the platypus, and the short- and long- beaked echidnas, and their distributions are limited to Australia and New Guinea. Apart for a short weaning period, milk is the sole source of nutrition and protection for the hatchlings which are altricial and immunologically naive. The duration of lactation in these mammals is prolonged relative to the gestational length and period of incubation of eggs. Much of the development of monotreme young occurs in the non-sterile *ex-utero* environment. Therefore the role of milk in the growth, development and disease protection of the young is of significant interest. By sequencing the cDNA of cells harvested from monotreme milk, we have identified a novel monotreme- specific transcript, and the corresponding gene was designated as the EchAMP. The expression profile of this gene in various tissues revealed that it is highly expressed in milk cells. The peptides corresponding to the EchAMP protein have been identified in a sample of echidna milk *In silico* analysis indicated putative antimicrobial potential for the cognate protein of EchAMP. This was further confirmed by *in vitro* assays using a host of bacteria. Interestingly, EchAMP did not display any activity against a commensal gut floral species. These results support the hypothesis of enhancement of survival of the young by antimicrobial bioactives of mammary gland origin and thus emphasize the protective, non- nutritional role of milk in mammals.

## Introduction

Monotremes represent a fascinating combination of both reptilian and mammalian characters: they lay shelled eggs while having a prototherian lactation process [1]. The prototherian and therian lineages appear to have diverged 166–220 million years ago and modern monotremes, which are confined to Australia and New Guinea, show a mixture of specialized adaptations and plesiomorphic features [2], [3], [4]. All three extant monotremes are highly specialized for their specific invertebrate diet: the semi-aquatic platypus feeds principally on benthic invertebrates, the short- beaked echidna feeds on ants, termites and pasture grub and the long- beaked echidna of New Guinea feeds on a variety of invertebrates in soil, leaf litter and rotting logs. As in marsupials, much of the development of the monotreme young occurs outside the mother’s body [5] and for the tiny, altricial hatchlings, apart for a short weaning period of about 12 days [6], milk is the only source of nutrition during the period of suckling, which is prolonged relative to gestation and incubation [7]. The role of milk in the growth, development and disease protection of young is yet to be established and there is speculation that the survival of eggs and the young of monotremes is enhanced by microbial inhibitors of cutaneous or mammary gland origin [8], [9]. Protective properties of milk in a vast range of mammals have been reported: for example, anti- parasitic activity of human milk lipase [10], antimicrobial activity of tammar wallaby milk cathelicidins and WFDC2 protein [11], [12], antifungal activity of bovine milk lactoferrin [13] and bacteriostatic activity of murine milk whey acidic protein [14]. In this study, for the first time, we identify a novel, monotreme-specific transcript that shows abundant expression in the milk cells during late- lactation. The peptides corresponding to the EchAMP protein have been identified in a sample of echidna milk. The cognate recombinant protein of EchAMP has displayed significant antibacterial activity against a host of bacteria, while showing no effect on a harmless gut commensal species. We suggest that this milk protein may have an important role in protecting the vulnerable monotreme young in the pouch and non-sterile burrow environments.

## Materials and Methods

### Ethics Statement

This work was carried out under permit from the Tasmanian Department of Primary Industries, Water & Environment, and the University of Tasmania Animal Ethics Committee, and through the University of Adelaide and research permits were provided by South Australian Department of Environment and Heritage and complies with the Australian Code of Practice for the Care and Use of Animals for Scientific Purposes (2004).

### Milk Collection and Isolation of Mammary Cells

Milk was collected from one echidna (*Tachyglossus aculeatus*) ‘Big Mamma’ at two time points within the same lactation period during late-lactation on 3^rd^ December 2004 (sample A1) and 27^th^ January 2005 (sample A2) on Kangaroo Island, SA, Australia. The samples were centrifuged at 2000 g for 5 minutes at 4°C to pellet cells and milk was removed. Cells harvested from sample A1 was used for the isolation of total RNA for subsequent cDNA library construction. Milk was collected from another lactating echidna (*Tachyglossus aculeatus,* Animal #4815) at two time points within same lactation period during late-lactation on 17^th^ January 2011 (sample B1) and 24^th^ January 2011 (sample B2) at Lovely Banks, a grazing property in the southern midlands, 55 km north of Hobart, Tasmania (longitude 147° 14′, latitude 42° 25′S), Australia. Prior to milk collection, the animals were anaesthetized and injected intramuscularly with 0.2 ml of synthetic oxytocin (2 IU, Syntocin, Sandoz-Pharma, Basel, Switzerland). The mammary glands were gently massaged, squeezed and milk was collected. Cells were removed from them as described before.

### Identification and Cloning of EchAMP Gene

EchAMP was found to be one of the novel, highly expressed transcripts as determined by the cDNA sequencing of echidna mammary cells [15]. Total RNA (0.6 ug) isolated from echidna milk cells was used to generate cDNA using the Clontech SMART PCR cDNA synthesis kit (Clontech, Sydney, New South Wales, Australia) [15]. EchAMP Primers (5′-CTGCATAAGCTTAGCGTGGATCTTGCCTCTGT-3′; 5′-GACTAGCTCGAGGTCTCTTTTGGATAAGAGGTTTGGA-3′ ) were designed to amplify the entire coding sequence using the Primer3 online tool (v.0.4.0). Sequences of restriction sites HindIII and XhoI were included in the forward and reverse primers respectively, to ease the cloning of EchAMP sequence in the subsequent steps. EchAMP sequence was amplified from echidna milk cell cDNA by polymerase chain reaction (PCR) by using Taq DNA polymerase (Bioserve). PCR was performed for 35 cycles with extension at 72°C for 1 minute and annealing at 58°C for 30 seconds in each cycle. The amplicon was run on a 1% agarose gel to confirm the size and then was purified using QIAquick gel extraction kit (Qiagen, Australia), following the manufacturer’s instructions. It was then ligated using T4 DNA ligase (New England Biolabs, USA) into HindIII and XhoI-digested c-Flag pcDNA3 vector (Invitrogen) and then transformed into competent DH5α cells. Positive clones were selected and verified by sequencing.

### In Silico Analysis of EchAMP Protein Sequence

*In silico* analysis of the EchAMP cDNA sequence revealed that it contained an open-reading frame and was capable of translating to a protein of 90 amino acids. SignalP 4.0 server was used to predict the presence of a signal sequence and location of signal peptide cleavage site in the EchAMP protein sequence [16].

Sequences of monotreme casein proteins: echidna CSN1 (GenBank Accession no. ACU25786), echidna CSN2 (GenBank Accession no. ACU25783), echidna CSN3 (GenBank Accession no. ACU25791), platypus CSN1 (GenBank Accession no. ACU25780), platypus CSN2 (GenBank Accession no. ACU25779), and platypus CSN3 (GenBank Accession no.ACU25793) were retrieved from the NCBI website. Multiple alignment of the signal peptides of these sequences along with that of the EchAMP protein sequence was carried out using ClustalW [17].

The InterPro scan sequence search online tool was used to determine the presence of any known domains and functional sites in the EchAMP protein [18]. Additionally, a search was carried using the default parameters of the BLAST tools BLASTN and TBLASTX for a matching sequence for the EchAMP nucleotide and protein respectively against all standard databases that were available on the Ensembl genome browser [19].

### Cationicity, Hydropathicity, Alpha Helicity and Post-translational Modifications of EchAMP Protein

The grand average of hydropathicity (GRAVY) of the EchAMP protein was determined by ProtParam software using the Kyte-Doolittle algorithm [20] available through the Expasy Proteomics server. Using ProParam, hydropathicity plot was generated with window size taken as n = 7. Alpha-helicity plot was generated by ProtScale using the Deleage and Roux scale [21] which was also available through the Expasy Proteomics server. NetOGlyc 3.1 server was used to predict the mucin type GalNAc O-glycosylation sites present in EchAMP protein [22].

### Tissue Expression Profile of EchAMP in Echidna

About 500 mg of frozen tissue samples of heart, ileum, jejunum, duodenum, stomach, liver, thyroid, spleen, kidney, testis and penis of a male echidna were used for total RNA extraction using Tripure Isolation reagent (Roche Diagnostics, Castle Hill, NSW, Australia) according to the manufacturer’s guidelines. RNA was also isolated from echidna milk cells, which represented the cells from the mammary gland during lactation. Concentrations of RNA were determined using a Nanodrop 2000 Micro-Volume UV-Vis Spectrophotometer (Wilmington, DE, USA). Reverse Transcription was performed using Superscript III™ First Strand Synthesis System (Invitrogen, Mount Waverly, Vic, Australia) with 1 µg of total RNA from above samples as templates. PCR was performed using EchAMP primers and GoTaq™ DNA polymerase (Promega). Amplification comprised of 30 cycles of 95°C for 30 seconds, 58°C for 30 seconds and 72°C for 1 minute and a final extension of 72°C for 5 minutes. The clone of EchAMP in c-Flag pcDNA3 plasmid was used as a positive control and the PCR mixture without the template DNA served as the negative control. To confirm the integrity of RNA and the first strand synthesis product, echidna GAPDH was amplified using the primers 5′-GACTCATGACTACAGTCCATGCCAT-3′ and 5′-GGACATGTAGACCATGAGGTCCAC-3′. The PCR products were checked on a 1.2% agarose gel and the DNA bands were visualized using SYBR safe staining under UV light.

### Identification of EchAMP Protein in Echidna Milk

Milk samples A1, A2, B1 and B2 were used for the visualization of total proteins by SDS- polyacryalmide gel electrophoresis. Total protein quantification of the samples was performed using the Micro BCA Protein assay Kit (Thermo Scientific, USA). 70 µg of each protein sample was electrophoresed for 3 hours at 100V using a 12% SDS-Polyacrylamide gel [23]. After staining the gel with Coomassie Blue, individual bands of protein sample B1 were excised from the gel and subjected to in-gel trypsin digestion [24]. Briefly, the excised bands were cut into tiny cubes and spun down in micro centrifuge tubes. The gel pieces were destained with 100 mM ammonium bicarbonate: acetonitrile (1∶1) solution and incubated with vortexing for 15 minutes. This step was repeated thrice to completely de-stain the gel pieces. They were then washed thrice with acetonitrile at room temperature with occasional vortexing until the gel pieces shrunk and became white. To remove any residual acetonitrile, the gel pieces were dried in a vacuum centrifuge. Enough trypisn buffer (10 mM ammonium bicarbonate containing 9% acetonitrile) was added to cover the gel pieces and the tubes were left at 4°C for 30 minutes. If needed, more trypsin buffer was added and then 10 µl of trypsin (10 µg/mL) was added to each tube. The tubes were kept at 4°C for about 30 minutes and then incubated at 37°C for 16 hours. Following this, the peptides were extracted twice by using 50% acetonitrile containing 5% trifluoroacetic acid. The collected peptides were dried in a vacuum centrifuge and processed further for identification by Mass spectrometry.

In order to desalt, concentrate and purify the peptides, 10 µl ZipTips (Merck Millipore) containing C18 resins were used according to the manufacturer’s instructions. The peptides were finally eluted with 8 µl of 60% acetonitrile containing 0.1% trifluoroacetic acid. They were subsequently dried and reconstituted in 15 µl of 5% acetonitrile containing 0.1% formic acid and 13 µl was loaded onto linear trap quadrupole (LTQ)-Orbitrap Velos instrument (Thermo Fisher Scientific). The software used for the analysis was Thermo Proteome Discoverer 1.3.0.339. A database containing the EchAMP protein sequence was loaded onto the SEQUEST algorithm and the raw data (spectra) obtained from the run was searched against this. Enzyme specificity was set to trypsin digestion with 2 missed cleavages and methionine oxidation as a dynamic modification. Peptide identification was accepted if they passed the filter criteria that was set to delta CN value 0.100, X corr vs charge values as 1.9 (+1 charge), 2.20 (+2 charge) and 3.10 (+3 charge) and protein probability as 0.001.

### Identification of EchAMP Gene in the Platypus Genome

The echidna EchAMP cDNA sequence was aligned using BLAST against the platypus genome (Ensembl Ornithorhynchus anatinus version 67.1) made available on the Ensembl server. The GENSCAN Web server was used to predict the locations and exon- intron structures of the orthologue of EchAMP gene on the platypus genome.

### Transfection of HEK-293T Cells and Collection of Conditioned Media

HEK-293T cells (Human embryonic kidney cell line) were maintained in DMEM high glucose media containing 10% FBS and incubated at 37°C under 5% CO_2_ condition. A day prior to transfection, HEK-293T cells (10^6^ cells per 35 mm dish) were seeded in serum-free Opti-MEM media. On the day of transfection, the media was replaced with 2 mL of fresh Opti-MEM media and the cells were transfected with Lipofectamine-2000 (Invitrogen) reagent following the manufacturer’s instructions. A pBS-KS vector containing a GFP (Green fluorescent protein) sequence cloned in-frame was used as a transfection control. The cells were transfected with vector c-Flag pcDNA3 containing no insert (empty vector) or the construct c-Flag pcDNA3-EchAMP. The success of transfection was verified using the fluorescent microscope to view the GFP-transfected cells. Media were collected 24 and 48 hours post transfection and centrifuged at 1000 rpm for 5 minutes to pellet the cells and debris. The resulting media supernatant were stored as conditioned media at −80°C until use.

To detect the presence of EchAMP protein in the conditioned media, a Criterion pre-cast denaturing, 15% polyacrylaminde gel (Biorad) was run by loading equal volumes of vector conditioned and EchAMP conditioned media collected 24 and 48 hours post transfection along with control HEK conditioned media, each mixed with protein loading dye. The gel was run under denaturing conditions at 200V for 1 hour. Following this, the protein bands were silver stained using the Pierce Silver Stain Kit (Thermo Scientific), following the manufacturer’s instructions.

In order to determine the presence of EchAMP protein in the respective conditioned media, Anti-Flag M2 Affinity Gel (Sigma Aldrich, USA) was used according the manufacturer’s instructions. Briefly, EchAMP conditioned media collected 48 hours post transfection was passed through the column to allow the binding of the Flag-tagged EchAMP protein to the resin. The column was washed thrice with Tris- buffered saline (50 mM Tris- Cl with 150 mM NaCl, pH 7.4) to remove any non-specifically bound proteins. Elution of the bound EchAMP protein was done by competitive elution with five one- column volumes of a Tris- buffered saline containing 100 µg/mL of FLAG peptide (Sigma Aldrich, USA). Samples from each stage of the purification procedure was run on a 15% SDS-Polyacrylamide gel and silver stained.

EchAMP conditioned media collected 48 hours post transfection was used for further assays.

### Antibacterial Assays

Antibacterial assays were performed using the alamarBlue cell viability reagent (Invitrogen), following the manufacturer’s instructions. All bacterial strains, *Escherichia coli* ATCC 2348/69, *Staphylococcus aureus* ATCC 29213, *Staphylococcus aureus* ATCC 25923, *Staphylococcus epidermidis*, *Salmonella enterica* ATCC 43971, *Pseudomonas aeruginosa* ATCC 27853 and *Enterococcus faecalis* ATCC 10100 were streaked on IsoSensitest (ISA) agar (Oxoid) plates and incubated overnight at 37°C. Isolated colonies of each strain were inoculated into 3 mL of IsoSensitest broth and grown overnight at 37°C with agitation in a shaker incubator. The overnight cultures were inoculated into fresh ISA broth at a ratio of 1∶100 and grown at 37°C with agitation until the OD_600_ = 0.6. An empirically determined count of 100 cells in 50 µL of ISA broth were added to each well of a 96-well black wall, clear bottomed plate (Costar; Corning Incorporated, Corning, NY, USA). Conditioned media (40 µL of EchAMP protein or pcDNA3 empty vector control) and alamarBlue (10 µL) were added to each well. Bacitracin (100 µg/mL) was used as the positive control for the assay. The plates were incubated at 37°C with shaking and fluorescence was measured every hour at an excitation of 544 nm and emission at 590 nm using Glomax Multi Detection System (Promega). All treatments were performed in triplicate and experiments were repeated at least thrice.

### Statistical Analysis

Statistical analysis of all comparative data was done using the two-tailed *t*-test, taking the statistical significance at *P*<0.05.

## Results

### Identification of EchAMP Transcript

**Figure 1:**
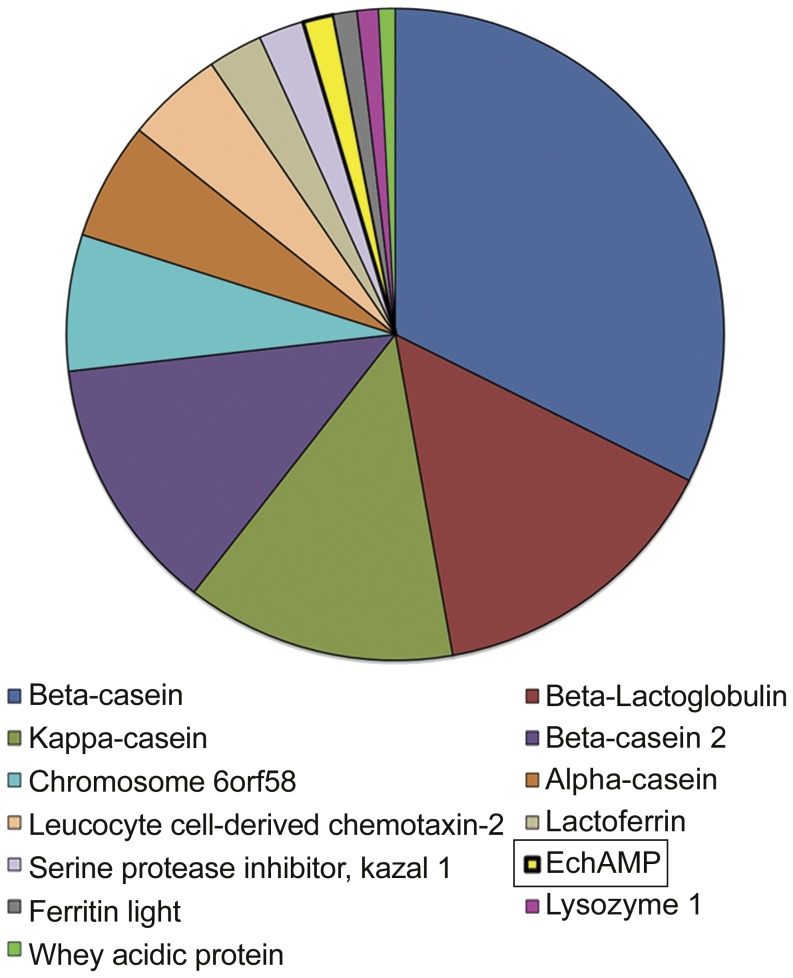
Identification of the EchAMP transcript. Relative abundance of the EchAMP transcript in echidna milk cells as compared to other major milk proteins. A novel sequence was found to be one of the highest expressed transcripts as determined by cDNA sequencing of echidna milk cells [15]; the corresponding gene was named as EchAMP (GenBank Accession no. KC148542).

Since the monotremes are protected species and access to their tissue samples is limited, a non- invasive approach for the analysis of their lactation was employed [15]. Echidna milk cells were harvested from milk collected during late- lactation. The cells were homogenized and processed for the isolation of purified total RNA, which was used as a starting material to generate an echidna cDNA library. The titre of the library was about 5.8×10^4^ cfu. Randomly picked clones were sequenced and relative gene expressions from EST counts were estimated. Briefly, of the 922 total EstID counts, a novel, un-annotated sequence appeared 13 times, being the highest after the sequences for *CSN2, BLG, CSN3, CSN2b, C6orf58, CSN1* and a few other known sequences (Figure 1). This novel sequence, which was the tenth most highly expressed transcripts was designated as the EchAMP sequence (GenBank Accession no. KC148542).

### Tissue Expression Profile of EchAMP in Echidna

**Figure 2:**
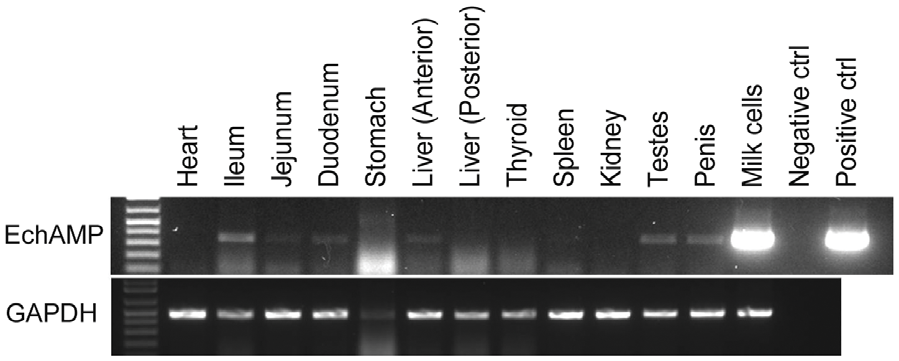
Expression profile of EchAMP in different echidna tissues. Expression of EchAMP relative to GAPDH as determined by Reverse- Transcriptase PCR in different echidna tissues. Abundant expression of EchAMP was seen in milk cells while a low level of expression was detected in intestine, liver, testes and penis.

The EchAMP gene expression was explored in different tissues of echidna. Reverse transcriptase PCR analysis for EchAMP gene relative to GAPDH showed that there was a high level of expression of EchAMP in the milk cells and a low level of expression in the intestine (comparatively higher in ileum than in jejunum and duodenum), liver, testes and penis. No expression was detected in the heart, thyroid, spleen and kidney (Figure 2).

### In-silico Analysis of EchAMP Protein

The EchAMP protein was predicted to contain a signal peptide of 19 amino acids**.** The most likely cleavage site was found to be between amino acids 19 and 20: ASG-AK. This cleavage site was in consensus with those of other eukaryotic secretory proteins.

**Figure 3:**
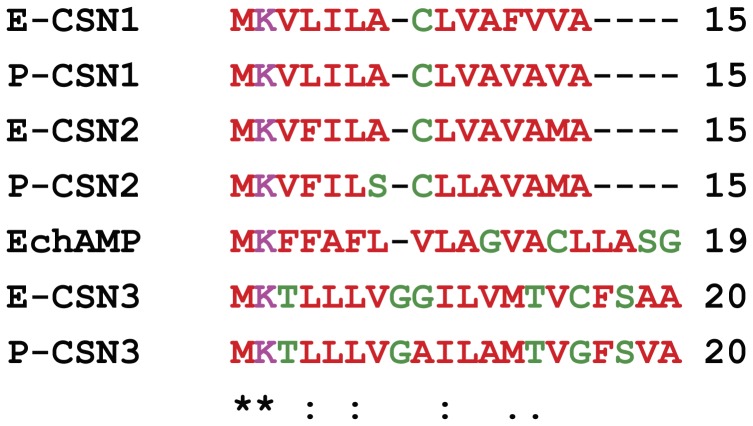
Multiple alignment of signal peptides of monotreme caseins and EchAMP protein. The EchAMP signal peptide shares two identical (*), three conserved (:) and two semi-conserved amino acids (.) with other monotreme casein signal peptides. E: Echidna; P: Platypus; CSN1: α-casein; CSN2: β-casein; CSN3: 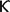-casein. Colors indicate the physicochemical properties of residues. Red: Small+Hydrophobic; Magenta: Basic; Green: Hydroxyl+Sulfhydryl+Amine.

Multiple alignment of signal peptides of monotreme casein proteins with that of EchAMP protein by ClustalW revealed that the EchAMP signal peptide shared two identical, three conserved, and two semi-conserved amino acids with the other monotreme casein signal peptides (Figure 3). These results provided substantial evidence for the EchAMP protein to be secretory in nature.

A search for the presence of any functional domains or repeats in the EchAMP protein sequence using the InterPro integrated database did not reveal any significant matches.

### The Grand Average of Hydropathicity (GRAVY) of EchAMP Protein

The EchAMP protein (with and without signal peptide) had a negative GRAVY score, indicating that it was hydrophilic in nature. This was in agreement with the respective percentages of cationic amino acid residues (Table 1).

**Table:** **Table1.** GRAVY of EchAMP protein.

Protein	GRAVY	No. of cationic residues	No. of anionic residues
EchAMP (full length; 90 aa)	−0.348	14	14
EchAMP (without signal sequence; 71 aa)	−0.977	13	14

GRAVY (grand average of hydropathicity) is the computed mean of hydrophobicity and hydrophilicity values for individual amino acid residues. A negative GRAVY indicates hydrophilicity while a positive value indicates hydrophobicity. aa: amino acids.

### Kyte and Doolittle Hydropathicity Plot of EchAMP Protein

**Figure 4:**
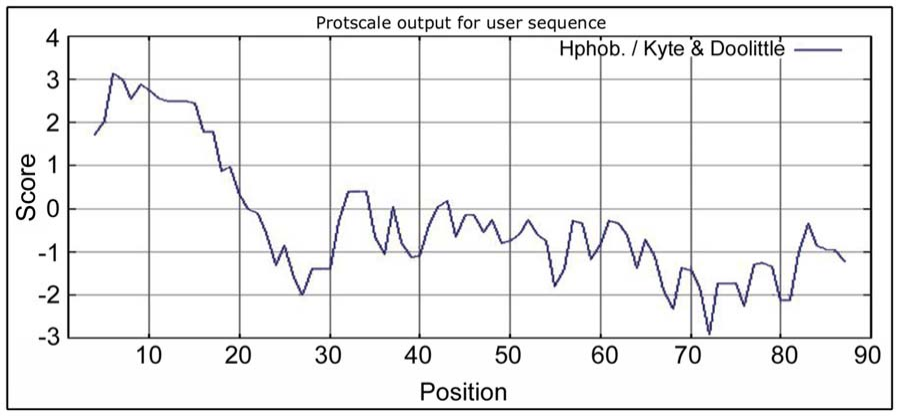
Kyte and Doolittle hydropathicity plot for EchAMP protein. Amino acid position is presented on the X-axis. Kyte and Doolittle hydropathicity scores (window size n = 7) for individual amino acids are on the Y-axis. The N-terminal region of EchAMP protein is hydrophobic while the central and C-terminal portions are hydrophilic. A steep increase in hydrophilicity is observed around the signal peptide cleavage site of the protein.

GRAVY score represents the average hydropathicity of the protein while Kyte and Doolittle plot presents the hydropathicity scores of individual amino acids and are calculated based on the neighbouring residues in a specified window size. Considering the window size n = 7, it was found that the N-terminal EchAMP protein was hydrophobic while the central and C-terminal portions were hydrophilic. A steep increase in hydrophilicity was observed around the signal peptide cleavage site of the protein (Figure 4).

### Identification of EchAMP Protein in Echidna Milk

**Figure 5:**
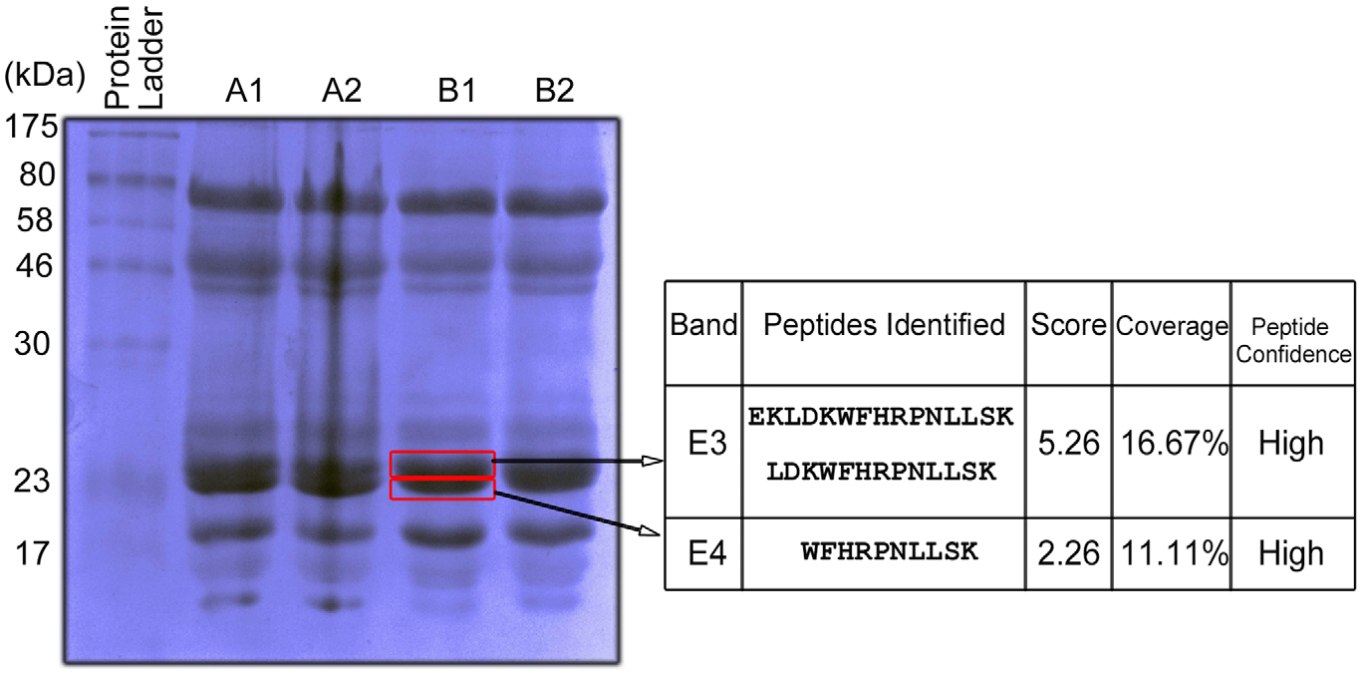
Identification of EchAMP protein in echidna milk. A1, A2 and B1, B2 represent milk samples collected from two lactating echidnas at two different time points during their late-lactation phase. 70 µg protein of each sample was electrophoresed for 3 hours at 100V using a 12% SDS-Polyacrylamide gel. Bands E3 and E4 were excised from the gel, subjected to in-gel trypsin digestion and anlaysed by LTQ Orbitrap Velos. The spectra of peptides from these bands showed a significant match with the predicted EchAMP protein with high confidence levels.

Four echidna milk samples (A1,A2, B1 and B2) collected from two individual animals at two different time points during late- lactation showed similar protein profiles on SDS-polyacrylamide gel (Figure 5). Identification of EchAMP protein was performed by Mass spectrometry of peptides obtained from bands corresponding to the expected EchAMP protein size. The spectra of peptides from bands E3 and E4 showed a significant match with the predicted EchAMP protein with high confidence levels.

### Deleage-Roux Alpha-helicity Plot for EchAMP Protein

**Figure 6:**
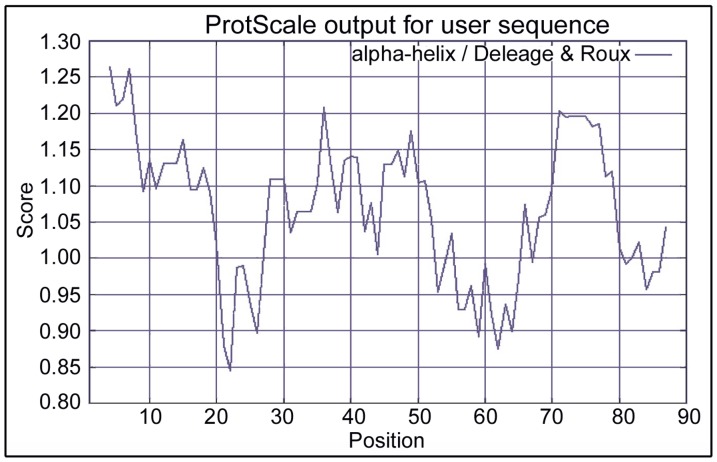
Deleage-Roux apha-helicity plot for EchAMP protein. The amino acid positions are indicated on the X-axis. The alpha-helicity scores are indicated on the Y- axis. The cut-off score was taken as 0.99. The EchAMP protein has significant alpha helical structure in the N-terminal region, followed by a steep decrease in the region spanning the amino acids 20–23. A second dip in alpha helicity is seen between the amino acids 55–65.

The alpha-helicity of EchAMP protein as determined using Deleage-Roux algorithm with a cut-off score of 0.99 showed that the protein had significant alpha helical structure in the N-terminal region, followed by a steep decrease in alpha helicity in the region spanning the amino acids 20–23. A second dip in alpha helicity was seen between the amino acids 55–65. The rest of the sequence was above the cut-off score although the middle hydrophilic region had the highest score (Figure 6).

### Post-translational Modifications for EchAMP Protein

**Figure 7:**
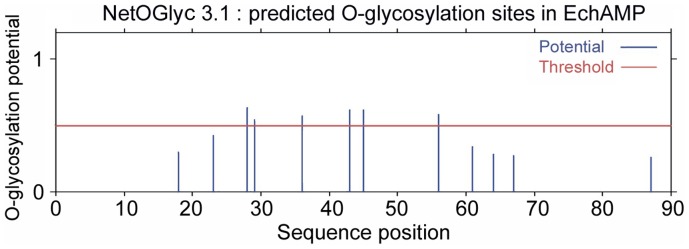
Mucin type O- glycosylation sites in EchAMP protein. NetOGlyc 3.1 server predicted the presence of six sites for mucin type O-glycosylation in EchAMP protein. The amino acid positions are indicated on the X-axis. The O-glycosylation potential is indicated on the Y-axis.

The predictions for mucin type GalNAc O-glycosylation sites in the EchAMP protein by NetOGlyc 3.1 server revealed that the protein had 6 potential sites for the same, well above the threshold (Figure 7).

### Identification of EchAMP Gene in the Platypus Genome

**Figure 8:**
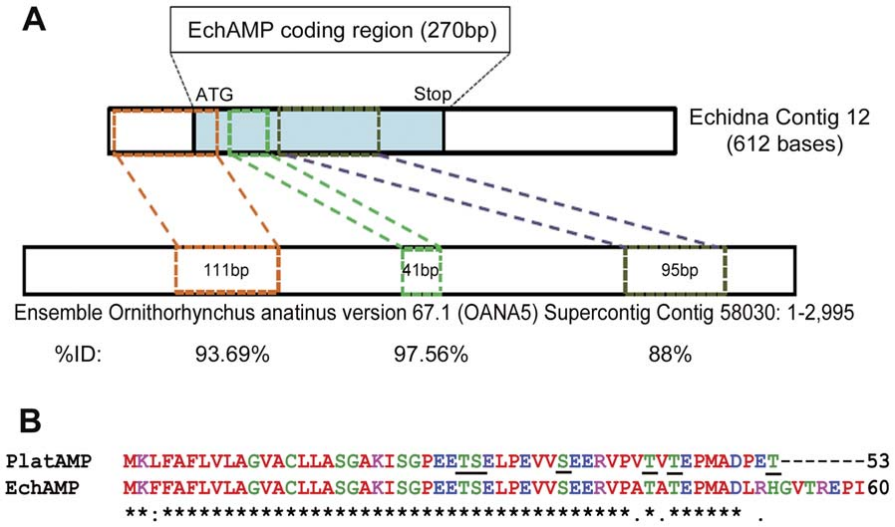
Identification of EchAMP gene in platypus genome. (**A**) Schematic representation of BLAST analysis of echidna EchAMP sequence against platypus genome. The sequences on platypus supercontig that showed homology with the echidna EchAMP were intervened with non-matching sequences and corresponded to exon and intron sequences respectively. The predicted exon/intron junctions in the platypus genome were in consensus with eukaryotic splice junctions. The last coding exon of the echidna EchAMP sequence did not show any homology to the platypus genome. The platypus orthologue of EchAMP was designated as PlatAMP. (**B**) Alignment of Genscan predicted partial PlatAMP peptide sequence with EchAMP protein sequence. The PlatAMP peptide shared 94% identity with the EchAMP protein. The underlined amino acids in the PlatAMP indicate the sites of potential mucin-type GalNAc O-glycosylation. Identical amino acid (*); conserved amino acid (:); semi-conserved amino acid (.). Colors indicate the physicochemical properties of residues. Red: Small+Hydrophobic; Magenta: Basic; Blue: Acidic; Green: Hydroxyl+Sulfhydryl+Amine.

Upon using the BLAST tool, the echidna contig of 612 bases containing the EchAMP cDNA sequence showed homology to three consecutive matching sequences in the Supercontig Contig 58030 of Ensemble Ornithorhynchus anatinus version 67.1 with percentage identities of 93.69%, 97.56% and 88.42% respectively (Figure 8A). The stretches of three consecutive matching sequences in the platypus genome were intervened with non-matching sequences and were predicted to correspond to exon and intron sequences respectively. The predicted exon/intron junctions of the EchAMP gene in platypus genome were in agreement with the eukaryotic splice junctions. However, the last coding exon of the echidna EchAMP transcript did not show any homology to the platypus genome as the platypus Supercontig did not extend into the 3′ region of the predicted platypus EchAMP gene. The orthologue of EchAMP gene on the platypus genome was designated as PlatAMP.

The Genscan predicted partial PlatAMP peptide sequence of 53 amino acids shared 94% identity with the EchAMP protein sequence and contained six potential sites for mucin-type GalNAc O-glycosylation (Figure 7B). The alpha-helicity plot of PlatAMP partial peptide as determined using Deleage-Roux algorithm was identical to that of the EchAMP protein (initial 53 amino acids), indicating the presence of significant alpha- helical structure.

Additionally, a search using the default parameters of the BLAST tools BLASTN and TBLASTX for a matching sequence for the EchAMP nucleotide and protein respectively against all standard databases available on the Ensembl genome browser did not yield any significant match.

### Transfection of HEK-293T Cells and Collection of Conditioned Media

**Figure 9:**
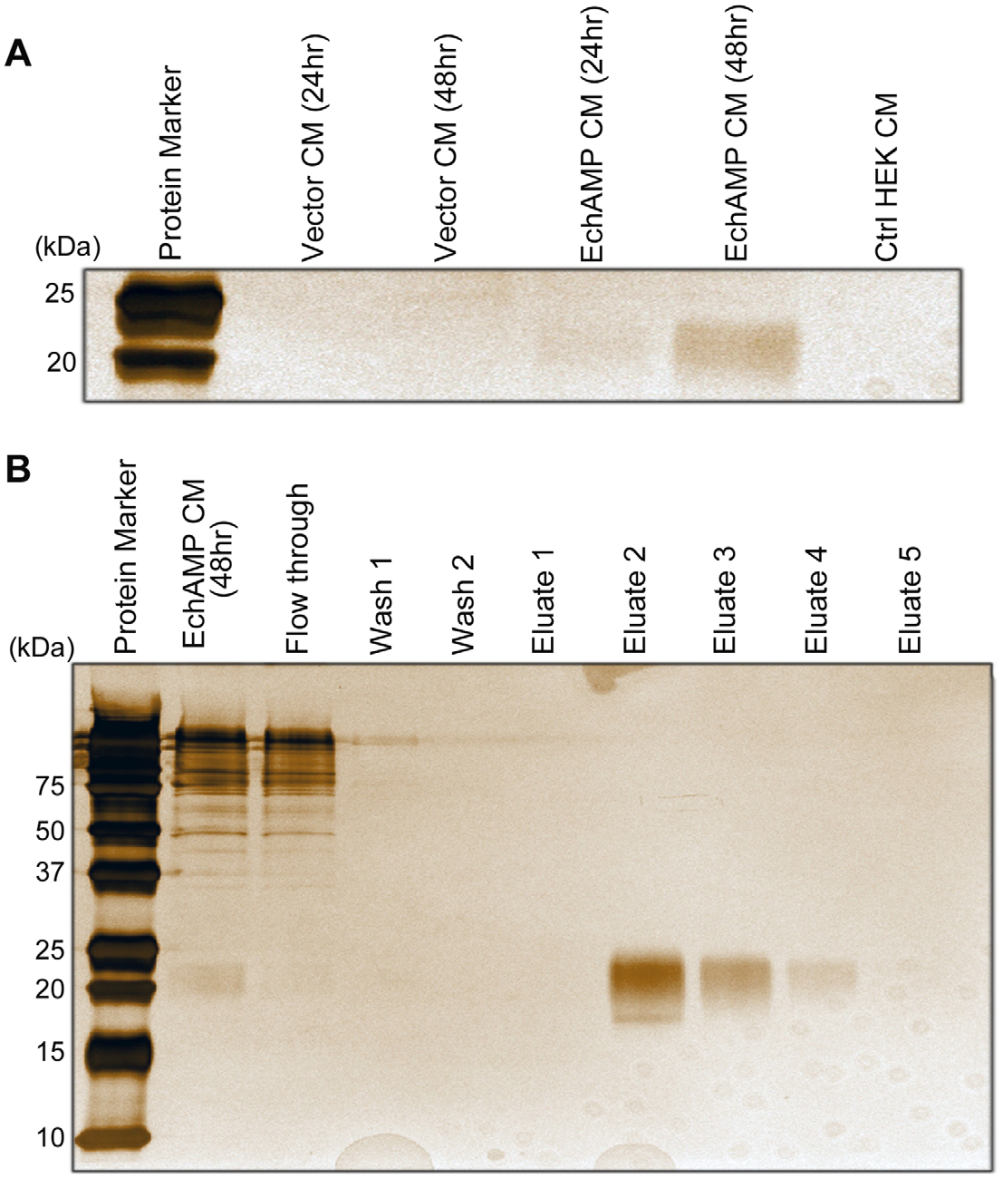
Detection of EchAMP protein in conditioned media. (**A**) The EchAMP protein present in conditioned media (CM) was detected by silver staining. No corresponding band was seen in vector conditioned or control (Ctrl) HEK293T conditioned media. The EchAMP protein was found to be higher in conditioned media collected 48 hours post transfection as compared to the one collected at 24 hours. (**B**) Purification of EchAMP protein using Anti-Flag M2 Affinity Gel. EchAMP protein was purified from the conditioned media collected 48 hours post transfection using an Anti- Flag M2 Affinity Gel column. Silver staining of samples from each stage of the purification procedure run on a 15% SDS- polyacrylamide gel show the presence of the purified EchAMP protein in the eluates.

In order to determine the function of the EchAMP protein, a recombinant protein was generated by transfecting HEK293T cells with c-Flag pcDNA3-EchAMP construct. Since the EchAMP protein was predicted to be of secretory nature, its secretion by the transfected cells into the surrounding media was determined. The EchAMP protein present in conditioned media was detected by silver staining of the polyacrylamide gel (Figure 9A). The EchAMP protein was found to be higher in conditioned media collected 48 hours post transfection as compared to the one collected at 24 hours. No corresponding band was seen in vector conditioned or control HEK293T conditioned media.

Further, the EchAMP protein was purified from the conditioned media collected 48 hours post transfection using an Anti- Flag M2 Affinity Gel column. Silver staining of samples from each stage of the purification procedure run on a 15% SDS- polyacrylamide gel showed the presence of the purified EchAMP protein in the eluates, confirming its presence in the respective conditioned media (Figure 9B).

### Antibacterial Assays: Bacteriostatic Effect of EchAMP Protein

**Figure 10:**
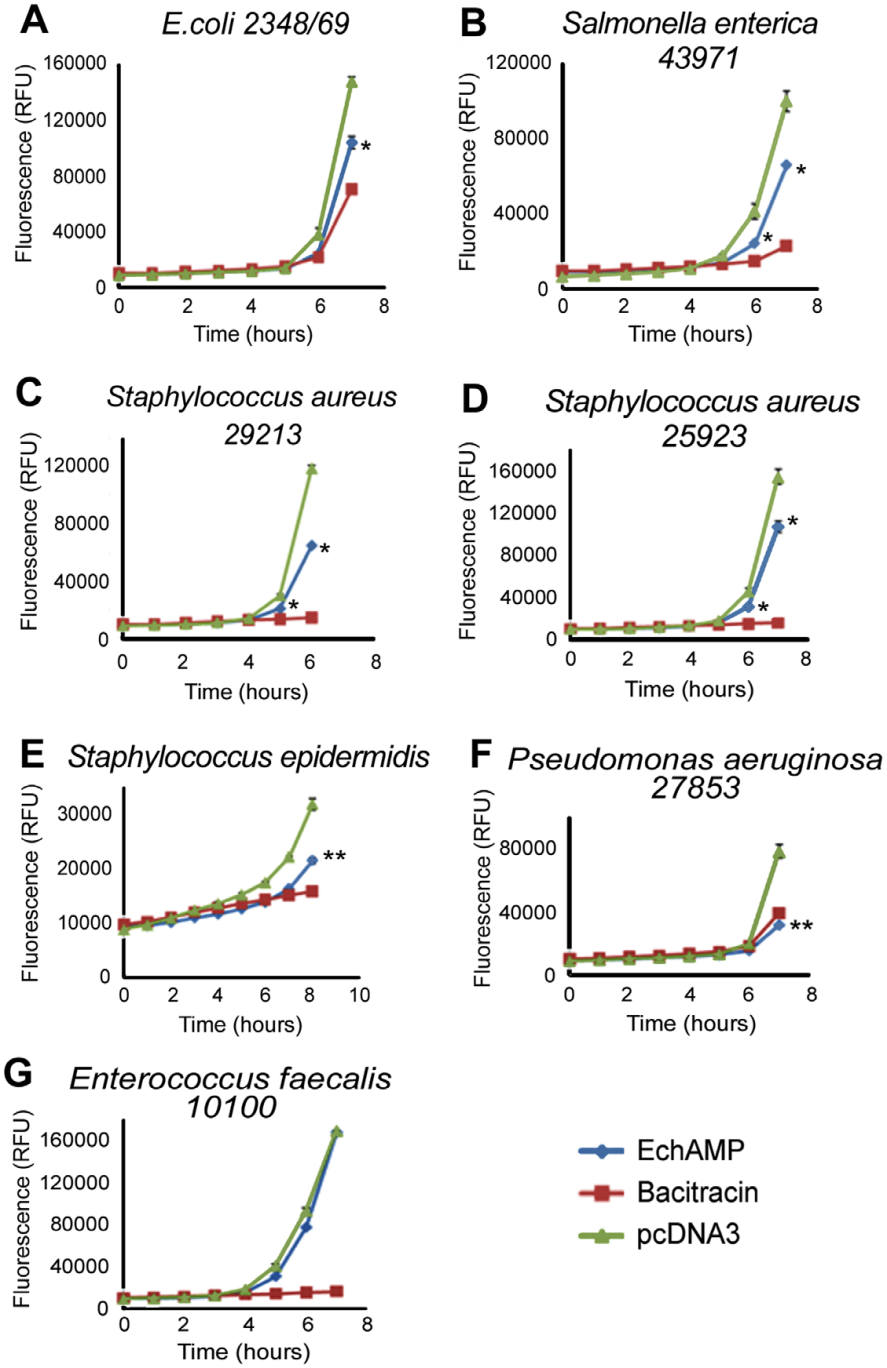
Antibacterial assays. (**A**) Bacteriostatic activity using *E.coli 2348/69*: EchAMP showed significant inhibition of growth as compared to the empty vector (pcDNA3) *P*<0.05 (**B**) Bacteriostatic activity using *Salmonella enterica 43971*: EchAMP showed significant inhibition of growth as compared to the empty vector (pcDNA3) *P*<0.05 (**C**) Bacteriostatic activity using *Staphylococcus aureus 29213*: EchAMP showed significant inhibition of growth as compared to the empty vector (pcDNA3) *P*<0.05 (**D**) Bacteriostatic activity using *Staphylococcus aureus 25923* : EchAMP showed significant inhibition of growth as compared to the empty vector (pcDNA3) *P*<0.05 (**E**) Bacteriostatic activity using *Staphylococcus epidermidis* : EchAMP showed highly significant inhibition of growth as compared to the empty vector (pcDNA3) *P*<0.05 (**F**) Bacteriostatic activity using *Pseudomonas aeruginosa 27853*: EchAMP showed highly significant inhibition of growth as compared to the empty vector (pcDNA3) *P*<0.05 (**G**) Bacteriostatic activity using *Enterococcus faecalis 10100*: EchAMP showed no inhibition of growth as compared to the empty vector and bacitracin *P>0.05* (* Statistically significant result *P*<0.05). Each assay was performed in triplicate and the experiments were repeated at least thrice. Standard error bars are indicated.

EchAMP protein was examined for inhibition of growth of a host of bacterial species (Gram positive bacteria: *Staphylococcus aureus* and *Enterococcus faecalis*; Gram negative bacteria: *Eischerichia coli*, *Pseudomonas aeruginosa* and *Salmonella enteric*a) (Figure 10). Antibacterial assays were performed using the conditioned media of HEK293T cells transfected with either EchAMP or empty vector pcDNA3. Antibacterial activity of EchAMP protein was compared to the no treatment control (empty vector) using a two-tailed t-test where P<0.05 was considered as significant.

EchAMP protein was found to have statistically significant bacteriostatic activity against *E. coli* (Figure 10A), *Salmonella enterica* (Figure 10B) and *Staphylococcus aureus* (29213 and 25923) (Figure10C and 10D). EchAMP showed statistically highly significant inhibition of growth of *Staphylococcus epidermidis* (Figure 10E) and *Pseudomonas aeruginosa* (Figure 10F) However, EchAMP showed absolutely no inhibition of growth of the bacterial species, *Enterococcus faecalis* (Figure 10G). Evidently, EchAMP exhibited strain-specific antibacterial activity.

## Discussion

In the current study, we have identified for the first time, a monotreme-specific transcript that is abundantly expressed in cells harvested from echidna milk at late- lactation and the protein product is secreted into the milk at this time. In order to study the function of the protein, we have expressed the EchAMP cDNA in HEK293T cells and the conditioned media from these cells displayed significant antibacterial activity against a host of Gram positive and Gram negative bacteria, confirming its predicted activity. Consecutive sequences showing homology to the EchAMP cDNA were found in the platypus genome, thereby indicating the possibility of its orthologue.

Harvesting cells from the milk of protected species such as monotremes has been pursued as a non-invasive approach for the analysis of their lactation [15]. These milk cells were an ambiguous mixture that could have included skin cells, immune cells, exfoliated epithelial cells from ducts and mammary or sebaceous glands. Presence of somatic cells in the milk of many other extant mammals such as sheep, cattle and humans have been reported [25], [26], [27]. In the current case, it was observed with cDNA sequencing that several casein and whey protein gene cDNAs were detected at high levels, indicating that monotreme milk cells harvested during peak lactation are enriched in exfoliated mammary epithelial cells. The sequence for EchAMP showed relatively high abundance, after the sequences for *CSN2, BLG, CSN3, CSN2b,* and *CSN1* which are major milk protein genes. Transcripts with relatively lower abundance than EchAMP were other major milk protein genes such as lysozyme and WAP suggesting that EchAMP plays a potentially prominent role during the lactation of echidna. The non-availability of cells or RNA from the mammary gland of a non-lactating echidna constrained us from determining the endogenous expression of EchAMP during the non-lactating period.

*In silico* analyses indicated that the cognate protein of EchAMP would be hydrophilic and secretory in nature. This was confirmed through the identification of peptides corresponding to EchAMP protein in a sample of echidna milk. Although monotreme milk has not been extensively studied, some of their main components have been described, such as caseins [15] and whey proteins including alpha- lactalbumin [28], [29], lysozyme [30], and WAP [31].

The discovery of three consecutive sequences in platypus genome showing homology to EchAMP cDNA with high percentage identity provided considerable evidence for the presence of orthologue of EchAMP gene in platypus, designated as PlatAMP. It is possible that the last coding exon of the echidna EchAMP transcript did not find any homology sequence in the platypus genome probably due to the latter’s incompleteness. Conversely, the variation of the sequences for the same gene among echidna and platypus, which are estimated to have shared the last common ancestor about 21.2 million years ago [32] cannot be ruled out. Insufficient platypus RNA samples curtailed further attempts to derive the matching sequence for the last coding part of the echidna EchAMP transcript by approaches such as PCR (Polymerase chain reaction) or RACE (Rapid amplification of cDNA ends). Nevertheless, it is noteworthy that the predicted PlatAMP partial peptide sequence of platypus was found to be highly similar (94%) to the EchAMP protein sequence and also shared similar features of alpha-helicity and post- translational modifications, indicating similar function. However, the expression of PlatAMP gene in the mammary/milk cells of platypus needs to be confirmed. With no significant matching sequence in any of the standard databases available at the Ensemble Genome Browser, we propose the EchAMP gene and hence its protein to be specific to monotremes alone.

Monotreme display a component of synapsidal reproduction by laying eggs that are incubated in the external, non-sterile environment and milk is the only source of nutrition during the period of suckling, which is prolonged relative to gestation and incubation [7], except for a short weaning period [6] It has often been speculated that during evolution, the protolacteal secretions enhanced the survival of eggs or the young by the virtue of their antimicrobial properties [9], [33], [34]. The same concept has been extended to monotremes, and it has been hypothesized that the survival of egg or the young is enhanced by microbial inhibitors of cutaneous or mammary gland origin [8]. These speculations led us to determine if a novel monotreme- specific milk protein such as EchAMP displayed any protective attributes. Indeed it appeared that EchAMP protein contained an overall significant alpha- helical structure, indicative of antimicrobial activity [35], [36]. In addition, the predictions from the Antimicrobial Peptide Database [37] for EchAMP protein sequence were suggestive of its possible antimicrobial potential by interacting with membranes by its virtue to form alpha helices (data not shown). Similarly, the predicted post-translational modification of the EchAMP protein, especially the occurrence of the mucin type GalNAc O-glycosylation sites also suggested that EchAMP protein may carry antimicrobial activity. In general, mucin type O-glycosylation has been structurally characterized for a number of tissue-specific secretions that includes mucins in milk from lactating breast epithelium [38], [39]. Also, with evidence such as milk mucin inhibiting the replication of rotavirus [40], it has been regarded that O-linked mucin carbohydrates may be one of the initial barriers belonging to the components of innate immunity [41]. It is also possible that the purpose of O-glycosylation is to shield the protein core against activity by proteases [42], thereby increasing its longevity.

The inhibition of bacterial growth *in vitro* during antibacterial assays using the EchAMP conditioned media provided confirmed evidence for the speculated antimicrobial activity of this protein. The EchAMP protein exhibited significant antibacterial activity against the pathogenic bacteria *E. coli, S. enterica, P. aeruginosa* and *Staphylococcus spp.* However, there was no antibacterial activity against the Gram positive commensal bacterium *Enterococcus faecalis*. This indicates that EchAMP protein targets specific-strains of bacteria. *Salmonellae, Eischerichia coli* and *Pseudomonas aeruginosa* are some of the bacteria that have been associated with infections in platypus, both in wild and in captivity [43]. Acute Salmonellosis has been reported in captive echidnas [44]. *Staphylococcus spp*. has been isolated from lesions in echidnas diagnosed with bacterial granulomata [44]. With respect to the mammary gland, mastitis is the most common infection and *Staphylococcus aureus, E. coli* and *Streptococcus spp*. have been frequently isolated in conditions of human and bovine mastitis [45], [46], [47], [48]. It has been reported that *E. faecalis* is one of the predominant harmless commensals found in the gastrointestinal tract in diverse species such as human, marsupials and most other vertebrates [49], [50], [51]. On the contrary, it has been shown that paucity of species in the sparsely colonized immature gut of infants, together with a lack of protective Gram positive species may aggravate the pathogenesis of neonatal necrotizing enterocolitis, because it may allow the overgrowth of pathogenic species [52], [53]. Correlating the faint expression of EchAMP transcript in the intestine and the EchAMP protein showing no antibacterial activity against *E. faecalis*, it may be appropriate to emphasize the specific antibacterial activity of this secretory milk protein against pathogenic bacteria while showing no activity on beneficial commensal species. During the weaning period, the gut flora in the young has to change from one adapted to a diet of highly digestible milk to one adapted to a diet of invertebrates. These microbes have to be passed on from the mother to the young and therefore it would seem reasonable that milk antimicrobials, produced by the mother, such as the EchAMP, would not have any effect on beneficial commensal species. As such, EchAMP bacteriostatic activity against *P. aeruginosa* and *S. epidermidis* is highly significant. *P. aeruginosa* is a versatile pathogen associated with a broad spectrum of infections in humans. It is an important cause of infection in immune-suppressed individuals and treatment is rendered increasingly problematic as the bacterium is inherently resistant to many antimicrobials and the resistance is being spread to few agents that remain as therapeutic options [54]. On the other hand, *S. epidermidis* was previously regarded as an innocuous commensal microorganism of skin and mucous membranes of human and other mammals [55], but now it is considered as an important opportunistic pathogen. Its specific molecular determinants that facilitate immune evasion, the presence of specific antibiotic resistance genes and hence its ability to cause chronic disease makes it extremely difficult to treat [56], [57]. It is generally seen that infections from yet another Gram positive bacterium, *S. aureus,* are usually caused from the same strain that the animal carries as a commensal. Such infections can affect the blood stream, skin, soft tissues and lower respiratory tracts [58], [59] and perhaps antibacterial proteins in monotreme milk such as the EchAMP confer protection to the young and the mammary gland of the mother against such infections. The Gram negative bacterium *S. enterica* is a highly host-adapted pathogen and its infections are a major problem in humans as well as in livestock animals such as cattle, pigs and chicken [60]. This bacterium is reported to contain several pathogenicity islands which encode virulence factors that induce inflammation in the host [61]. However, the bacterium is able to exploit the same inflammation for nutrients and outcompetes other bacterial species in the gut [62]. The other Gram negative bacterium *E. coli* belongs to a group of enteropathogens that exploit host epithelial cells and are the major cause of infantile diarrhea [63], [64].

Taken together, it is evident that EchAMP, a novel monotreme- specific milk protein is capable of conferring protection to the underdeveloped, immunologically naïve young outside the sterile confines of the uterus, in the harsh pathogen-laden environments. However, with the limitation of access to monotreme milk samples across the lactation period, we are unable to deduce any specific changes in expression of EchAMP during the lactation period. The monotreme mammary gland lacks nipples, and therefore the altricial young is more likely to ingest pathogens while suckling compared to any other species. This is a significant difference between monotremes and marsupials which also give birth to altricial young, but the milk delivery is aided by a nipple to which the young is attached continuously for the first 100 days of lactation [65], [66]. We propose that the evolution of nipples and development of offspring *in utero* in the placental mammals (Metatherian and Eutherian) could have led to the loss of selective pressure for the preservation of this gene and hence its subsequent disappearance in these species. For an example, there is a precedence of selective loss of genes involved in gastric function in platypus, which diverged from the Therian lineage early during mammalian evolution. Not with-standing the high conservation in vertebrates for more than 400 million years, genes encoding the gastric proteases, hormone gastrin, both the subunits of the gastric H^+^/K^+^-ATPase and the neurogenin-3 transcription factor have either been deleted or inactivated in platypus genome, giving rise to physiological differences in digestion between monotremes and therians [67].

### Conclusions

Monotremes are potential sources to discover new antimicrobials because they lay eggs and their subsequent development into young, all occur in the non-sterile, *ex-utero* environment. For the first time, we have identified a novel transcript that is specific to monotremes and is abundantly expressed in milk cells during late- lactation. The peptides corresponding to this transcript have been identified in echidna milk. Conditioned media from HEK293T cells expressing the EchAMP protein has been shown to display antibacterial activity against a host of Gram positive and Gram negative bacteria, while no activity was detected against a commensal gut floral species. Our data support the hypothesis of enhancement of survival of monotreme young by antimicrobial bioactives of mammary gland origin [8], [9]. Apart for a short weaning period of about 12 days during which the young echidna begins feeding outside the burrow but still takes milk from the mother [6], milk is the sole source of nutrition for the altricial hatchling during the period of suckling which is prolonged compared to gestation and incubation of eggs [7]. The *ex-utero* environment of the developing young is favorable for microbial attack and as the monotreme mammary gland has no nipple, the young is more likely to ingest microbial pathogens than the pouch young of marsupials which are attached to the nipple. During evolution, this would have favored the incorporation of antimicrobial agents into the glandular secretions in order to protect the young [34]. Our data are consistent with the speculation that monotreme genomes have evolved under evolutionary pressure to protect immunologically naïve young with broad spectrum antibiotics [68] and further emphasize an important, non- nutritional role of monotreme milk.
