# Correction: Identification and Functional Characterization of a Novel Monotreme- Specific Antibacterial Protein Expressed during Lactation

**DOI:** 10.1371/annotation/c5ad793d-aa77-4522-8e6b-3e77c89d0726

**Published:** 2013-06-19

**Authors:** Swathi Bisana, Satish Kumar, Peggy Rismiller, Stewart C. Nicol, Christophe Lefèvre, Kevin R. Nicholas, Julie A. Sharp

The accession number was omitted from the Results. The following sentence belongs after the sixth sentence of the first paragraph in the section: "The nucleotide sequence data reported for PlatAMP are available in the Third Party Annotation Section of the DDBJ/EMBL/GenBank databases under the accession number TPA: BK008723." 

